# Mingmu Xiaomeng Tablets Restore Autophagy and Alleviate Diabetic Retinopathy by Inhibiting PI3K/Akt/mTOR Signaling

**DOI:** 10.3389/fphar.2021.632040

**Published:** 2021-04-13

**Authors:** Yuwei Fang, Kangpei Shi, Haining Lu, Lin Lu, Bo Qiu

**Affiliations:** ^1^ Department of Ophthalmology, The Second Clinical College of Guangzhou University of Chinese Medicine, Guangzhou, China; ^2^ State Key Laboratory of Ophthalmology, Zhongshan Ophthalmic Center, Sun Yat-sen University, Guangzhou, China

**Keywords:** Mingmu Xiaomeng tablets, autophagy, diabetic retinopathy, PI3K/Akt/mTOR, inflammation

## Abstract

**Objective:** To investigate the effect of Mingmu Xiaomeng tablets (MMXM) on the expression of phosphoinositide 3-kinase (PI3K)/Akt/mammalian target of rapamycin (mTOR)-related proteins in a diabetic rat model.

**Methods:** Thirty-two male Sprague Dawley rats were randomly divided into four groups: normal control (NC), diabetic model (DM) control, MMXM, and calcium dobesilate (CD) Rats injected with streptozotocin (STZ) were used as an experimental diabetes model. After 14 weeks, autophagy and PI3K/Akt/mTOR signaling pathway proteins were detected by western blot. Glial fibrillary acidic protein (GFAP) expression in Müller cells was examined by immunohistochemistry. Retinal function was evaluated with electroretinography, and retinal ultrastructure was observed by transmission electron microscopy. Serum cytokine levels were detected with protein chip technology.

**Results:** MMXM restored autophagy by decreasing the protein expression of LC3-II and p62 and reducing the phosphorylation of PI3K, Akt, and mTOR, thus promoting autophagy. MMXM decreased GFAP expression in retinal Müller cells; restored electrophysiology indexes and retinal ultrastructures; and reduced serum levels of interleukin (IL)-1β, IL-4, IL-6, tumor necrosis factor-α, and vascular endothelial growth factor.

**Conclusion:** MMXM may protect the diabetic retina by inhibiting PI3K/Akt/mTOR signaling and enhancing autophagy.

## Introduction

Diabetic retinopathy (DR) is one of the main retinal vascular complications of diabetes and a major cause of visual impairment and blindness in working age people worldwide. According to the 9th edition of the global diabetes map released by the International Diabetes Federation in 2019, global adult population (20–79 years) prevalence is estimated to be 9.3% (463 million people) (Saeedi et al., 2019). The number of adult diabetes patients in China is as high as 116 million, ranking the first in the world and accounting for more than 1/4 of the total global number (Saeedi et al., 2019). The prevalence rates of DR, non-proliferative DR (NPDR), and proliferative DR (PDR) in Chinese patients with diabetes are 18.45, 15.06 and 0.99% respectively, with the highest and lowest incidences in southcentral and northwest China, respectively (Song et al., 2018). How to effectively treat DR is a hot topic in ophthalmology because it has great significance for preventing blindness.

Hyperglycemia-induced DR pathogenesis is related to four main biochemical changes: polyol pathway activation, increased advanced glycation end-products (AGEs), activation of protein kinase C, and induction of the hexosamine pathway (Wu et al., 2018; Shafabakhsh et al., 2019). These pathways induce inflammation, oxidative stress, and vascular dysfunction in the retina. DR can be divided into NPDR or PDR (Solomon et al., 2017). NPDR is characterized by microaneurysms, intraretinal hemorrhage hard exudates, and cotton wool spots. In PDR, neovascularization results in vitreous and retinal hemorrhages that can lead to retinal detachment; neovascularization in the iris leads to neovascular glaucoma that is a hallmark of the disease (Wilkinson et al., 2003). Recent studies have emphasized that inflammation, oxidative stress and autophagy are the pathogeneses of DR (Rosa et al., 2016; Dehdashtian et al., 2018; Cecilia et al., 2019; Sinclair and Schwartz, 2019; Kinuthia et al., 2020). Decreased and increased autophagy have been implicated in DR deterioration and alleviation, respectively (Fu et al., 2016).

Autophagy is an evolutionarily conserved catabolic pathway that controls cell homeostasis by disposing of cytoplasmic cargo, denatured proteins, damaged organelles, and invasive pathogens through lysosomal degradation (Mizushima and Komatsu, 2011). Balanced synthesis and degradation of cellular proteins and organelles maintains cell survival and homeostasis. Autophagy failure may lead to the accumulation of harmful damaged organelles and protein aggregates (Grumati et al., 2010). PI3K/Akt/mTOR signaling plays critical roles in regulating cell survival, differentiation, proliferation, and migration (Yu and Cui, 2016). Previous study indicates PI3K/Akt/mTOR signaling pathway is hyperactivated in human cancers (Ersahin et al., 2015; Tewari et al., 2019). New studies have found that PI3K/Akt/mTOR proteins are widely expressed in the retinal tissue of diabetic rats (Wang et al., 2019; He et al., 2020). Therefore, inhibition of PI3K/Akt/mTOR is a target for the treatment of diabetic retinopathy.

Mingmu Xiaomeng tablets (MMXM) are a hospital preparation developed in the early 1990s based on the clinical experience of Professor Meifang Zhang, a famous ophthalmologist at the Guangdong Provincial Hospital of traditional Chinese medicine (TCM), after several generations of clinical verification in ophthalmology. MMXM are mainly composed of modified Shengmai Powder and Wendan Decoction, and are widely used in patients with difficult ophthalmic diseases with qi-yin deficiency and phlegm-stasis syndrome (Zhan et al., 2012; Wang et al., 2014; Qin et al., 2019; Yan and Qin, 2019). Shengmai Powder can replenish qi, nourish yin, stimulate saliva, and reduce thirst; Wendan Decoction can regulate qi and phlegm, clean the gallbladder, and replenish the spleen; Tribulus terrestris and Buddleja officinalis Maxim soothe the liver and improve eyesight; and *Ilex* pubescens and Concha arcae can dissipate and disperse phlegm. Therefore, MMXM can be used to treat DR and improve the quality of life of affected patients.

Calcium dobesilate (calcium 2,5-dihydroxybenzenesulfonic acid; CD) was registered by the European Pharmacopoeia and British Pharmacopoeia in 1997 and 1998, respectively (Zhang et al., 2015). It is considered an angioprotective agent with anti-inflammatory, antioxidative, and anti-angiogenic properties (Wang et al., 2019) and prescribed as an alternative therapy for the treatment of diabetic retinopathy and other vascular disorders (Berthet et al., 1999; Rabe et al., 2011). CD is indicated and approved for the treatment of DR in several countries, and it is beneficial in the early stages of DR (Simó et al., 2015). For this reason, CD was chosen as a positive control drug in the present study.

Based on preliminary experiments and the existing literature (Zhang et al., 2011; Wang et al., 2018), we established a streptozotocin (STZ)-induced diabetic rat model, activated the PI3K/Akt/mTOR signaling pathway with the aim of triggering the onset of diabetic retinal changes. We hypothesized that MMXM would block the PI3K/Akt/mTOR signaling pathway and attenuate diabetic retinopathy, thus providing a scientific basis for the treatment of DR.

## Materials and Methods

### Drugs

MMXM was composed of Dang Shen [Codonopsis pilosula (Franch.) Nannf.], Mao Dong Qing (*Ilex* pubescens Hook. & Arn.), Wa Leng Zi (Concha arcae), Mai Dong [Ophiopogon japonicus (Thunb.) Ker Gawl], Chen Pi (Citrus reticulata Blanco, Pericarpium Citri Reticulatae), Zhi Shi (Citrus aurantium L. Aurantii Fructus Immaturus), San Qi [Panax notoginseng (Burkill) F.H. Chen], Ji Li (Tribulus terrestris L.), Mi Meng Hua (Buddleja officinalis Maxim.), Wu Wei Zi [Schisandra chinensis (Turcz.) Baill.] at a dry-weight ratio of 6:6:6:3:2:2:2:2:2:1. MMXM was provided by the Guangdong Provincial Hospital of TCM (Lot no. Z20080115, Guandong province, China). Calcium dobesilate capsules were purchased from Ebewe Pharma GmbH (Cat. No. H20140641, Unterach, Austria). Betaine, caffeic acid, hyperoside, notoginsenoside R1, hesperidin, ginsenoside Rb1, luteolin, quercetin, and ferulic acid were obtained from the National Institutes for Food and Drug Control (Beijing, China), chlorogenic acid was obtained from Solarbio Science and Technology Co., Ltd. (Beijing, China), and berberine was obtained from Aladdin Bio-Chem Technology Co., Ltd. (Shanghai, China).

### Chemical Analysis of Mingmu Xiaomeng by Ultra-high Performance Liquid Chromatography-Quadrupole/Orbitrap High Resolution Mass Spectrometry

Ground MMXM powder (200 mg) and 1 ml methanol:water (2:8, V:V) were vortexed to mix, centrifuged at 4°C for 10 min (14,000 rpm) and the supernatant was passed through a 0.22 μm filter membrane. The 11 standards were diluted to 2.00 mg/ml with methanol. All analyses were performed on Ultimate 3000 UPLC system (Thermo Fisher, Waltham, MA, United States) and Q Exactive mass spectrometer (Thermo Fisher). Both the positive and negative ion modes were applied in parallel reaction monitoring at a resolving power of 17,500 (scan range: 50–500 m/z). Mobile phases 0.1% formic acid aqueous solution (A) and acetonitrile (B) were used to separate the analytes on a Welch RP-C18 column (150 mm × 2.1 mm, 1.8 μm). The gradient elution was as follows: 0–0.5 min, 2% B; 0.5–6.5 min, 2%–98% B; 6.5–9.0 min, 98% B; 9.0–9.3 min, 98%–2% B; 9.3–10.0 min, 2% B. The flow rate was 0.3 ml/min, the injection volume was 5 μL, and the ion spray voltage was 3.2 kV. The aux gas heater and capillary temperatures were 350 and 300°C, respectively.

### Rats and Ethics Statement

**FIGURE 1:**
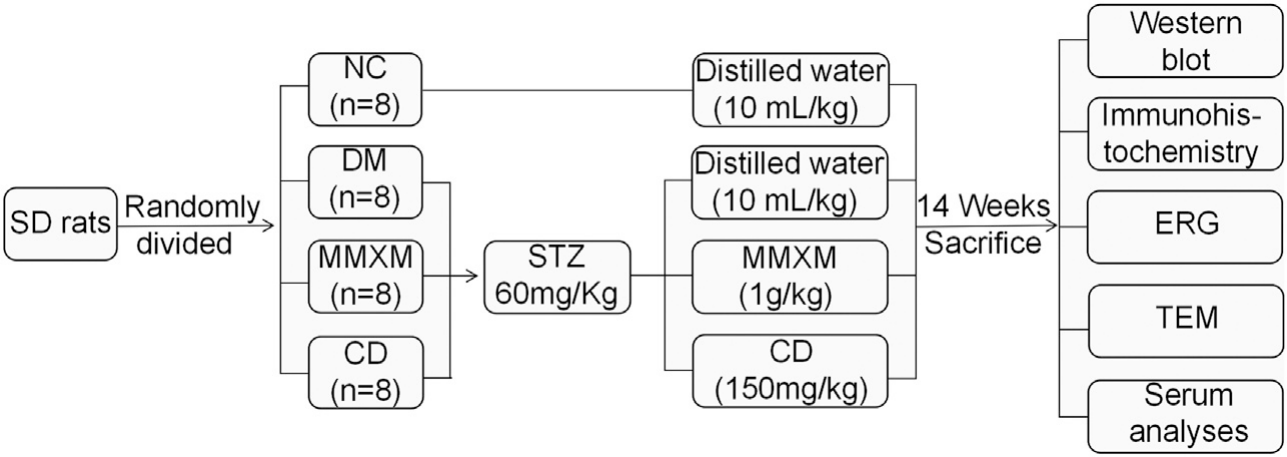
Animal experimental procedure.

The procedure of animal experiment is illustrated in Figure 1. Thirty-two male Sprague Dawley rats, specific pathogen-free grade, aged from 6 to 8 weeks old, were purchased from Beijing Si bei fu Biotechnology Co., Ltd [Certificate No. SCXK (Beijing) 2016-0002]. All rats were raised in the specific pathogen-free facility of the Animal Laboratory of the State Key Laboratory of Ophthalmology, Zhongshan Ophthalmic Center, Sun Yat-sen University. All procedures and animal care were carried out in accordance with the guidelines of the Institute of Vision and Ophthalmology on the use of animals for research and were approved by the Animal Ethical Committee (Permit Number 2018–031). All rats were housed at room temperature (22–24°C) under 12 h light-dark cycles with 40–60% relative humidity and had free access to standard food and water ad libitum.

### Animal Model and Treatments

After 1 week of adaptive feeding, rats were fasted for 12 h prior to STZ (Sigma Co., Ltd. St. Louis, MO, United States) injection. DM was induced by intraperitoneal injection of STZ dissolved in 0.1 mol/L of citrate buffer (pH 4.5) at a dose of 60 mg/kg body weight. Age-matched control rats received an equal volume of vehicle. 3 days after STZ injection, a random glucose level >16.7 mmol/L was considered diabetic (Liu et al., 2020). The animals were divided into four groups (*n* = 8/group) as follows: 1) normal control rats (NC) treated with distilled water (10 ml/kg); 2) STZ-induced diabetic rats (DM) treated with distilled water (10 ml/kg); 3) STZ-induced diabetic rats treated with MMXM at 1 g/kg (MMXM) (Selection basis is based on the supplementary material, 1 g/kg was the lowest dose that was statistically significant when compared with DM group. *p* < 0.05, Supplementary Figure S1); and 4) STZ-induced diabetic rats treated with calcium dobesilate capsules at 150 mg/kg (CD). The MMXM and CD doses were based on the daily doses commonly prescribed in humans.

### Western Blot

Retinal tissues were prepared with radioimmunoprecipitation assay lysis buffer containing phenylmethanesulfonyl fluoride and phosphate inhibitor (100:1:1). Lysates were centrifuged at 12,000 r/min for 30 min at 4°C, and the supernatants were collected. Equal amounts of protein (20 µg) were separated by sodium dodecyl sulfate polyacrylamide gel electrophoresis and transferred to polyvinylidene difluoride (PVDF) membranes (0.45 µm, Millipore, Billerica, MA, United States). After blocking with 5% non-fat dry milk at room temperature for 2 h, the PVDF membranes were washed with Tris-buffered saline containing 0.1% Tween-20 (TBST) three times and incubated overnight at 4°C with different primary antibodies (anti-phosphorylated [p]-phosphoinositide 3-kinase [PI3K], 1:500; anti-PI3K, 1:1,000; anti-p-Akt, 1:1,000; anti-Akt, 1:1,000; anti-p-mammalian target of rapamycin [mTOR], 1:1,000; anti-mTOR, 1:1,000; anti-LC3B, 1:1,000; anti-p62/SQSTM1, 1:1,000. anti-glyceraldehyde 3-phosphate dehydrogenase [GAPDH], 1:1,000). The above-mentioned primary antibodies were obtained from Cell Signaling Technology (CST, Danvers, MA, United States) except p-PI3K which was from Bioworld (Bloomington, MN, United States). Next, the PVDF membranes were washed with TBST three times and incubated with horseradish peroxidase (HRP)-conjugated secondary antibodies (CST) at room temperature for approximately 1 h. Finally, the PVDF membranes were washed with TBST three times, and the blots were developed with chemiluminescence reagent using an enhanced chemiluminescence kit (Advansta, San Jose, CA, United States).

### Immunohistochemistry

The eyeballs were immersed in Formaldehyde, Aceticacid, Alcohol, Saline (FAS; Wuhan Servicebio Technology Co., Ltd., Wuhan, China), dehydrated in alcohol, and then embedded in paraffin. Paraffin-embedded tissue blocks were cut into serial 4 μm sections. Paraffin-embedded retina sections were dewaxed, rehydrated, washed with phosphate-buffered saline (PBS), incubated with 3% H_2_O_2_ to block endogenous peroxidase activity, and blocked with 3% bovine serum albumin. They were then incubated with GFAP antibody (Abcam, Cambridge, United Kingdom) at a dilution of 1:1,500 overnight at 4°C. The next day, the sections were washed with PBS three times for 5 min each and incubated with an HRP-conjugated secondary antibody (K5007, DAKO, Glostrup, Denmark) for 50 min at room temperature. After washing with PBS, the sections were stained with 3,3-diaminobenzidine tetrahydrochloride for approximately 1 min, counterstained in hematoxylin, dehydrated in absolute alcohol, cleared in xylene, and finally mounted in synthetic resin for microscopic examination.

### Electroretinography

After 12 weeks of treatment, ERG was performed according to the International Society for Clinical Electrophysiology guidelines (McCulloch et al., 2015). Before testing, rats were dark-adapted for 12 h and anesthetized intraperitoneally with 10% chloral hydrate (3.5 ml/kg) under a dim red light. Next, 1% tropicamide was used to dilate the pupil. Tetracaine hydrochloride eye drops were administered to anesthetize the cornea, and hydroxypropylmethylcellulose eye drops were used to keep the cornea moist. The active electrode was a ring placed at the center of the cornea. The reference and ground leads were placed in the subcutaneous space of the cheek and the tail, respectively. A Roland visual electrophysiological RETIport/scan 21 recorder instrument (Roland Consult GmbH, Wiesbaden, Germany) was used to detect and record the experimental data.

### Transmission Electron Microscopy

The eyes were enucleated, fixed in 2.5% glutaraldehyde for 1 h at room temperature, trimmed into 1 mm^2^ pieces, then fixed in 2.5% glutaraldehyde solution overnight at 4°C. The next day, the retinas were dehydrated in a graded series of ethanol baths and embedded in Epon 812. The site of the retina studied with TEM was selected by toluidine blue staining. Ultrathin sections were cut with a Leica UC7 microtome (Leica, Wetzlar, Germany), stained with uranyl acetate and lead citrate, and examined under a Hitachi HT7700 electron microscope (Hitachi, Tokyo, Japan).

### Serum Analyses

Serum was collected from the upper aorta and centrifuged. Samples were analyzed and quantified with multianalyte arrays (QAR-CYT-3 Quantibody array; Raybiotech, Norcross, GA, United States).

### Statistical Analysis

All data are presented as mean ± standard error of the mean. Differences between groups were analyzed using Statistical Package for the Social Sciences version 22.0 (SPSS 22.0, IBM Corp. Armonk, NY, United States). Results were analyzed using one-way analyses of variance for comparisons of more than three groups, followed by Least Significant Difference post hoc multiple comparisons. *p* < 0.05 was considered statistically significant. Graphical results were analyzed using GraphPad Prism 7 (GraphPad Software, Inc. La Jolla, CA, United States).

## Results

### Quantification of the 11 Compounds in Mingmu Xiaomeng

**FIGURE 2:**
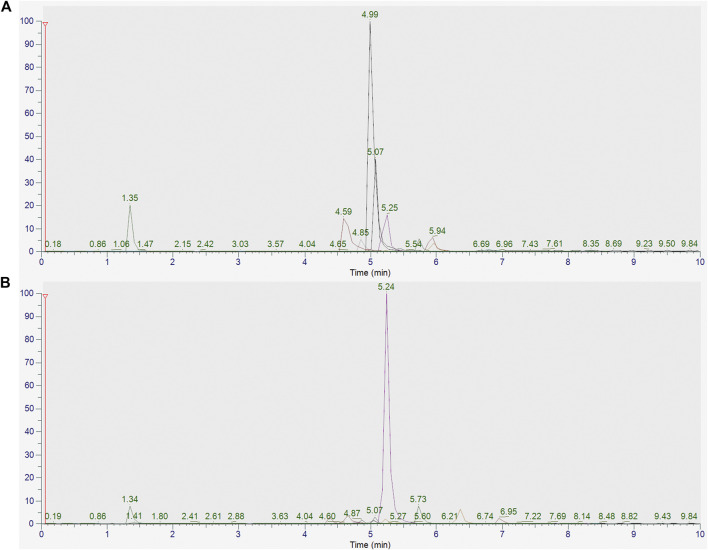
**(A)** UPLC-Q-Orbitrap HRMS analysis of standards (betaine, chlorogenic acid, caffeic acid, berberine, hyperoside, notoginsenoside R1, hesperidin, ginsenoside Rb1, luteolin, quercetin, and ferulic acid). **(B)** UPLC-Q-Orbitrap HRMS analysis of MMXM.

The peaks in the UPLC profile were identified as betaine, chlorogenic acid, caffeic acid, berberine, hyperoside, notoginsenoside R1, hesperidin, ginsenoside Rb1, luteolin, quercetin, and ferulic acid (Figure 2). The contents of these constituents in MMXM were determined to be 38.15, 26.33, 26.89, 1.19, 9.53, 31.21, 488.43, 146.23, 27.38, 1.47, and 8.56 μg/g, respectively, by UPLC-Q-Orbitrap HRMS (Table 1).

**TABLE 1:** Standard spectrum information and contents of the 11 compounds in MMXM.

Name	CAS	Formula	Molecular Weight	Mode	Parent	Product	RT (min)	Mass Concentration (μg/g)
Betaine	107–43-7	C5H11NO2	117.15	+	118.09	59.07	1.35	38.15
Caffeic acid	327–97-9	C16H18O9	354.31	+	355.10	163.04	4.66	26.33
Chlorogenic acid	331–39-5	C9H8O4	180.15	+	181.05	163.04	4.85	26.89
Berberine	2086–83-1	C20H18NO4	336.12	+	336.12	320.09	4.99	1.19
Hyperoside	482–36-0	C21H20O12	464.38	+	465.10	303.05	5.07	9.53
Notoginseno-side R1	80,418–24-2	C47H80O18	933.13	−	933.54	423.36	5.15	31.21
Hesperidin	520–26-3	C28H34O15	610.56	+	611.20	303.09	5.25	488.43
Ginsenoside Rb1	14,197–60-5	C42H72O13	784.30	+	1,109.61	325.11	5.73	146.23
Luteolin	491–70-3	C15H10O6	286.23	+	287.06	153.02	5.94	27.38
Quercetin	117–39-5	C15H10O7	302.00	+	303.05	229.05	5.98	1.47
Ferulic acid	1,135–24-6	C10H10O4	194.19	+	195.06	177.05	6.80	8.56

### Effect of Mingmu Xiaomeng on PI3K/Akt/mTOR Regulation of Autophagy in Diabetic Rats

**FIGURE 3:**
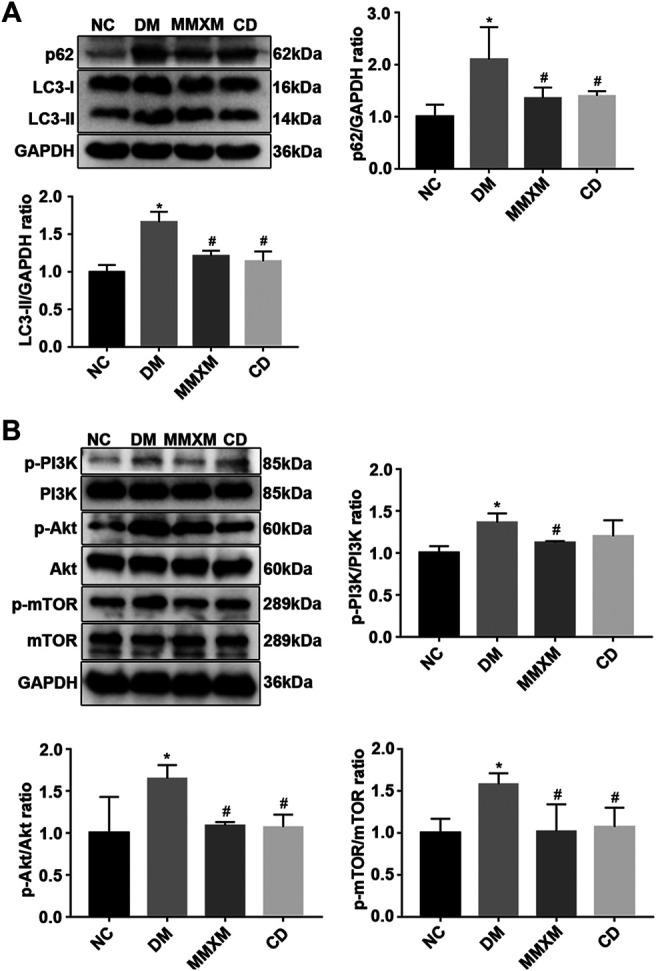
**(A)** Relative expressions of p62 and LC3-II protein in retinal tissues. **(B)** Relative expression of p-PI3K/PI3K, p-Akt/Akt, p-mTOR/mTOR protein in retinal tissues. *p < 0.05 vs. NC; #*p* < 0.05 vs. DM.

Compared with the NC group, LC3-II and p62 expression were significantly increased in the retina of the DM group (*p* < 0.05, Figure 3A). Compared with the DM group, LC3-II and p62 levels in the MMXM and CD groups significantly decreased (*p* < 0.05). The expression levels of p-PI3K, p-Akt and p-mTOR were significantly upregulated in the MMXM and CD groups versus the NC group (*p* < 0.05, Figure 3B). Compared with the DM group, p-PI3K, p-Akt, and p-mTOR protein levels were significantly downregulated in the MMXM group (*p* < 0.05), while only p-Akt and p-mTOR protein levels were significantly lower in the CD group (*p* < 0.05).

### Effect of Mingmu Xiaomeng on Glial Fibrillary Acidic Protein Expression in Retinal Müller Cells

**FIGURE 4:**
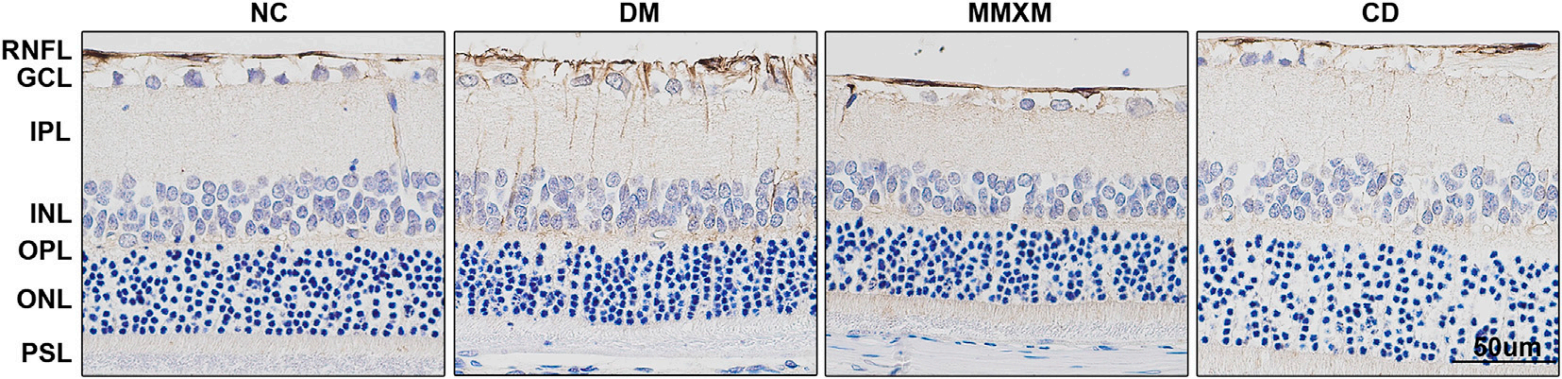
GFAP expression of retinal Müller cells by immunohistochemistry. GCL, ganglion cell layer; INL, inner nuclear layer; IPL, inner plexiform layer; OPL, outer plexiform layer; ONL, outer nuclear layer; PSL, photoreceptor segment layer, RNFL, retinal nerve fiber layer. Original magnification 400×. Positive cells were stained brownish yellow. Scale bar: 50 μm.

GFAP positivity in the nerve fiber, ganglion cell, and inner reticulum layers were significantly enhanced in the DM group compared to the NC group (Figure 4). Long brown processes penetrated the inner and outer nuclear layers, and there was a trend of increased GFAP expression throughout the entire optic layer. Compared with DM group, the numbers of GFAP brown filaments were decreased in the nerve fiber and ganglion cell layers in the MMXM and CD groups.

### Effect of Mingmu Xiaomeng on Electroretinography Results in Diabetic Rats

**FIGURE 5:**
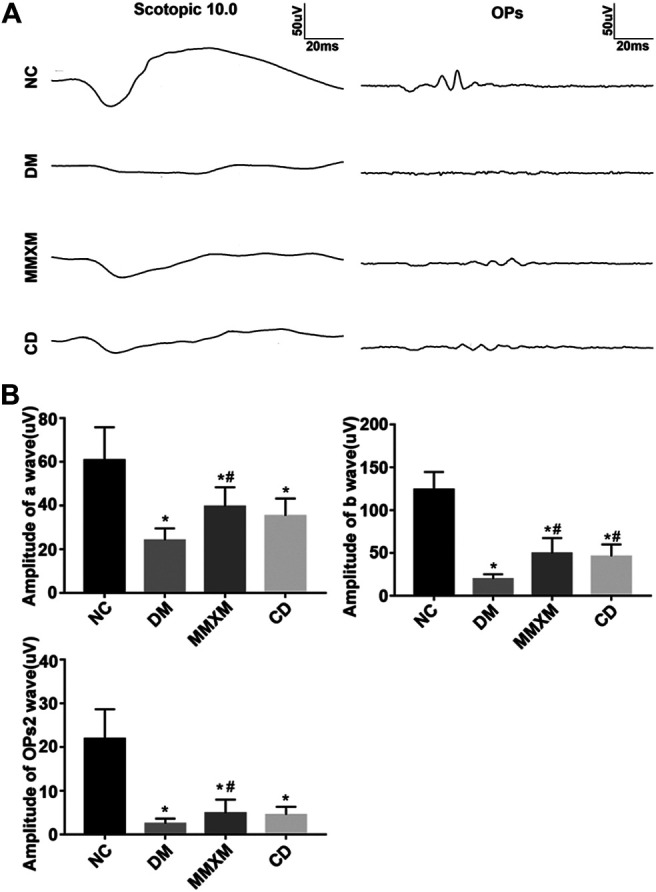
**(A)** Typical scotopic ERG traces. **(B)** The amplitude statistics of a, b, and OPs2 waves. Values are presented as mean ± SD.*p < 0.05 vs. NC; #*p* < 0.05 vs. DM.

Compared with the NC group, the amplitudes of the a, b, and the second wave of oscillatory potentials (OPs2) decreased in DM rats; the amplitude of the b wave was more affected than that of the a wave, but the differences for both were statistically significant (*p* < 0.05, Figure 5). Compared with the DM group, the amplitudes of the a, b, and OPs2 waves in the MMXM group increased after gavage, but the differences were only significant for the a and b waves (*p* < 0.05). The amplitudes of all three waves increased in the CD group, but only the b wave amplitude difference was statistically significant (*p* < 0.05). Collectively, these results indicate that MMXM restored retinal function in diabetic rats.

### Effect of Mingmu Xiaomeng on Retinal Ultrastructure

**FIGURE 6:**
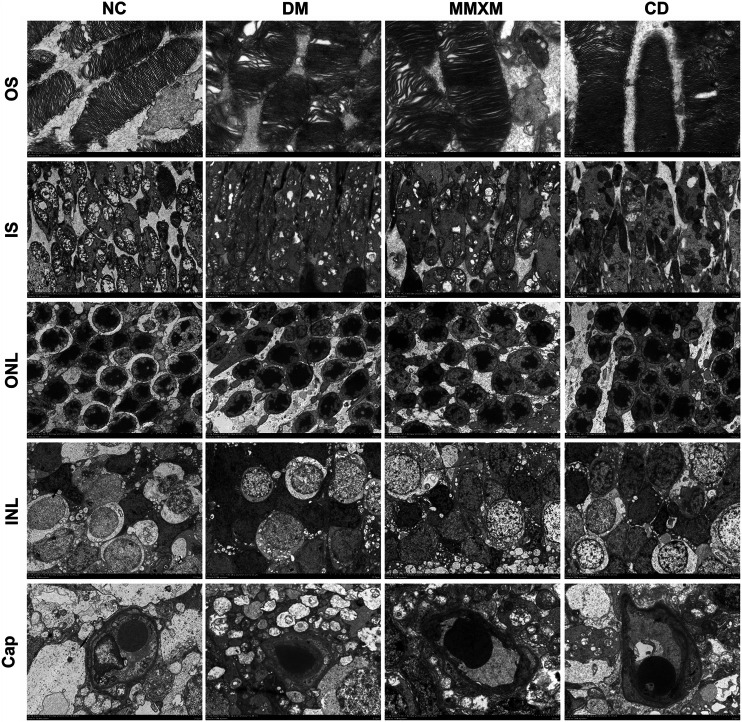
Retinal cell ultrastructure. OS: outer segment, Original magnification 6,000×; IS: inner segment, original magnification 2,500×; ONL: outer nuclear layer, original magnification 1,000×; INL: inner nuclear layer, original magnification 1,000×; Cap: capillary, original magnification 2,500×.

The NC group had a normal retina pattern, while the outer segments in the DM group were shorter and showed a disorganized inner structure including swelling, degeneration, and vacuoles (Figure 6). There were some giant mitochondria with obvious vacuolation; the outer nuclear layer was disordered, the nuclear membranes were sunken, and the intercellular space was evident. The electron density of Müller cells increased, and mitochondria swelled and vacuolated in the cytoplasm around the nuclei. The basement membranes of capillaries were thickened with narrow lumens. In the MMXM and CD group, the outer segment membrane discs of photoreceptor cells were arranged in an orderly manner, the local area was loose, and there was no obvious mitochondrial swelling or vacuole-like changes. The outer nuclear layer was more orderly, but there was some mitochondrial edema and vacuolation in the cytoplasm around the nuclei of Müller cells in the nuclear layer; the basement membranes of capillaries were uneven.

### Effect of Mingmu Xiaomeng on Serum Cytokine Levels

**FIGURE 7:**
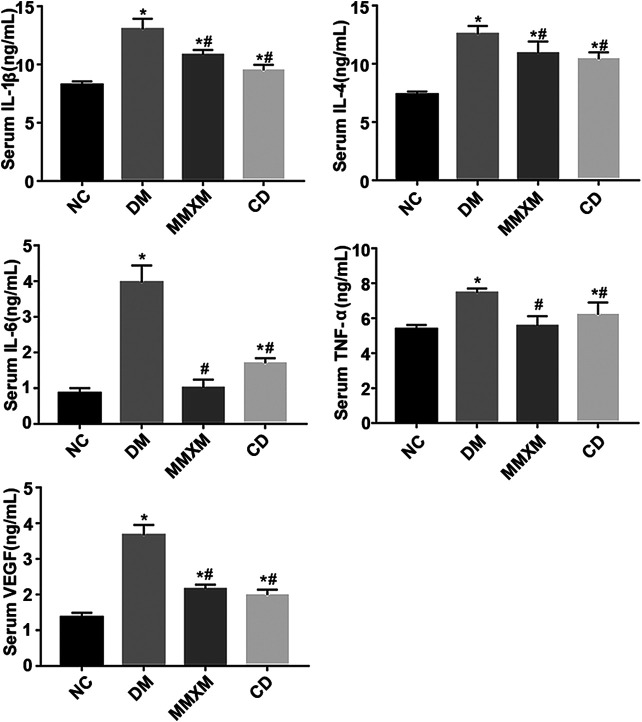
Serum IL-1β, IL-4, IL-6, TNF-α, and VEGF levels.*p < 0.05 vs. NC; #*p* < 0.05 vs. DM.

Compared with the NC group, interleukin (IL)-1β, IL-4, IL-6, tumor necrosis factor (TNF)-α, and vascular endothelial growth factor (VEGF) expression in the DM group were significantly increased (all *p* < 0.05, Figure 7). Compared with the DM group, serum IL-1β, IL-4, IL-6, TNF-α, and VEGF levels decreased significantly in the MMXM group (all *p* < 0.05), as did serum IL-1β, IL-4, IL-6, TNF-α, and VEGF levels in the CD group (all *p* < 0.05).

## Discussion

Autophagy functions as a double-edged sword in DR and may have a role in its initiation and exacerbation. Autophagic activity can promote cell survival under mild stress, while dysregulated autophagy results in cell death during severe stress (Dehdashtian et al., 2018). LC3-II is a marker of autophagosome formation (Kameyama et al., 2017; Yao et al., 2017); it can accumulate due to increased upstream autophagosome formation or impaired downstream autophagosome-lysosome fusion. To distinguish between these possibilities, we examined the autophagic substrate p62/SQTSM1, a selective substrate of autophagy (Huang et al., 2016). To further clarify the effect of MMXM on the diabetic rat retina, we examined components of the upstream PI3K/Akt/mTOR pathway. PI3K/Akt/mTOR signaling is very important in autophagy regulation; it also plays critical roles in regulating cell survival, differentiation, proliferation, and migration (Yu and Cui, 2016). mTOR is a key regulator in the initiation phase of autophagy and can also inhibit autophagy after activation (Noda, 2017; Rabanal-Ruiz et al., 2017). PI3K and Akt are upstream of mTORC1 and activate mTOR to inhibit autophagy (Cong et al., 2016).

We assessed the effect of MMXM on autophagy regulation via the PI3K/Akt/mTOR signaling pathway by western blot. Compared with the NC group, LC3-II and p62 levels in the DM group were increased, as were phosphorylated proteins in the PI3K/Akt/mTOR signaling pathway. This indicated that DM activated retinal autophagy, while PI3K/Akt/mTOR signaling inhibited excessive autophagy. Compared with the DM group, LC3-II, p62, p-PI3K, p-Akt, and p-mTOR protein levels were decreased in retinal tissue in the MMXM group. These results suggest that MMXM effectively inhibited activation of the PI3K/Akt/mTOR signaling pathway, promoted lysosome fusion, and significantly enhanced autophagy.

Müller cells account for 90% of mammalian retinal glial cells. They form supporting structures that radiate across the retina and participate in outer and inner limiting membrane formation (Coorey et al., 2012). This structure helps regulate the physiological and pathological responses of the retinal vascular system and neurons. There is growing evidence to suggest that retinal neurodegeneration precedes overt microvascular changes characteristic of DR (Simó and Hernández, 2012; Simó and Hernández, 2014). Müller cells are key players in the pathogenesis of retinal neurodegenerative diseases since they have a variety of supporting functions to maintain RGC homeostasis (Toft-Kehler et al., 2017). GFAP is a specific glial cell marker that is often used to assess the response of glial cells under pathological conditions. Upregulated GFAP expression in Müller glial cells is one of the early symptoms of retinal metabolic stress and has been reported in animal models and tissue from diabetic patients with no to mild NPDR (Mizutani et al., 1998; Abu-El-Asrar et al., 2004).

In this study, retinal GFAP expression was detected by immunohistochemistry. GFAP immunopositivity in normal rat retinas mainly appeared in the nerve fiber and ganglion cell layers. In diabetic rats, GFAP-positive staining of the entire retina was significantly enhanced, especially in the nerve fiber, ganglion cell, inner plexiform, inner nuclear, and outer plexiform layers. GFAP-positive staining of the MMXM and CD groups was weaker than the DM group but stronger than the NC group. Our results confirm that retinal glial cells changed in the early stage of diabetes. Specifically, we observed reactive activation of glial cells (mainly Müller cells), which indicated that they were damaged due to abnormal glucose metabolism. MMXM inhibited GFAP overexpression in retinal Müller cells in diabetic rats and protected against early diabetic retinal neurodegeneration.

We found that the amplitudes of a, b, and OPs waves decreased in DM rats, which indicated that they had developed DR (Zhang et al., 2018). The a wave reflects photoreceptor function, the b wave reflects bipolar and Müller cell function, and the OPs wave indirectly reflect function of retinal blood vessels (Nagai et al., 2017). OPs2 is a typical representative of OPs waves and can reflect the overall level of this response category (Zhang et al., 2018). After 3 months of MMXM treatment, retinal function was improved for all three waves compared with the untreated DM group. This is the first reported evidence that MMXM could rescue retinal function and protect retinal blood vessel function, and was associated with suppression of aberrant GFAP expression in Müller cells.

A growing body of evidence suggests that local inflammatory processes in the retina are indispensable for early DR development (Joussen et al., 2004; Gerhardinger et al., 2009). Müller cells are an important source of many growth factors and cytokines when stimulated by stress conditions (Zong et al., 2010). Müller glial cells are also closely related to DR as they can induce the expression of acute-phase response proteins and other inflammation-related genes (Lorenzi and Gerhardinger, 2001). Inflammation causes greater permeability; leukostasis; and local increases in inducible nitric oxide synthase, cyclooxygenase-2, intracellular adhesion molecule (ICAM)-1, nuclear factor (NF)-κB, caspase 1, VEGF, nitric oxide, prostaglandin E2, IL-1β, and other cytokines (Kern, 2007).

The PI3K/Akt/mTOR pathway is a convergence of proinflammatory mediators, growth factors, and downstream substrates that regulate cell survival processes. This makes inhibition of PI3K/Akt/mTOR signaling an attractive therapeutic target for DR. mTOR inhibitors could suppress the pro-inflammatory phenotype. Dampening mTOR signaling could also decrease NF-κB expression, thus reducing the expression of downstream proinflammatory mediators such as TNF-α, monocyte chemoattractant protein-1, VEGF, IL-1β, RAGE, vascular cellular adhesion molecule-1, and ICAM-1 (Jacot and Sherris, 2011). These pro-inflammatory cytokines, chemokines, and adhesion molecules have been demonstrated to play roles in DR development and progression (Adamiec-Mroczek et al., 2010; Roy et al., 2017).

## Conclusion

In this study, activation of the PI3K/Akt/mTOR signaling cascade and decreased autophagy activity in diabetic rats promoted the expression of various inflammatory cytokines and diabetic retinal neurodegeneration. After intragastric MMXM administration, inflammatory factor expression decreased, autophagy was restored and retinal function was improved, which may be related to inhibition of the PI3K/Akt/mTOR pathway. This study provided the first evidence that MMXM directly contribute to the prevention of the early DR. Future *in-vitro* study is required to explore the effects of MMXM or its various components on retinal cells induced by high glucose, and will help to elucidate the mechanisms underlying treatment of DR. It will be desirable to assess the preparation at a lower dose in order to assess the therapeutic relevance of the results further.

## Data Availability

The raw data supporting the conclusions of this article will be made available by the authors, without undue reservation, to any qualified researcher.

## References

[B1] Adamiec-Mroczek J. Oficjalska-Młyńczak J. Misiuk-Hojło M. (2010). Roles of endothelin-1 and selected proinflammatory cytokines in the pathogenesis of proliferative diabetic retinopathy: Analysis of vitreous samples. Cytokine 49 (3), 269–274. 10.1016/j.cyto.2009.11.004 20015663

[B2] Berthet P. Farine J. C. Barras J. P. (1999). Calcium dobesilate: Pharmacological profile related to its use in diabetic retinopathy. Int. J. Clin. Pract. 53 (8), 631–636. 10692760

[B3] Cecilia O.-M. José Alberto C.-G. José N.-P. Ernesto Germán C.-M. Ana Karen L.-C. Luis Miguel R.-P. (2019). Oxidative stress as the main target in diabetic retinopathy pathophysiology. J. Diabetes Res. 2019, 8562408. 10.1155/2019/8562408 31511825 PMC6710812

[B4] Cong J. Liu R. Wang X. Jiang H. Zhang Y. (2016). MiR-634 decreases cell proliferation and induces apoptosis by targeting mTOR signaling pathway in cervical cancer cells. Artif. Cell Nanomedicine, Biotechnol. 44 (7), 1694–1701. 10.3109/21691401.2015.1080171 26367112

[B5] Coorey N. J. Shen W. Chung S. H. Zhu L. Gillies M. C. (2012). The role of glia in retinal vascular disease. Clin. Exp. Optom. 95 (3), 266–281. 10.1111/j.1444-0938.2012.00741.x 22519424

[B6] Dehdashtian E. Mehrzadi S. Yousefi B. Hosseinzadeh A. Reiter R. J. Safa M. (2018). Diabetic retinopathy pathogenesis and the ameliorating effects of melatonin; involvement of autophagy, inflammation and oxidative stress. Life Sci. 193, 20–33. 10.1016/j.lfs.2017.12.001 29203148

[B7] El-Asrar A. M. A. Dralands L. Missotten L. Al-Jadaan I. A. Geboes K. (2004). Expression of apoptosis markers in the retinas of human subjects with diabetes. Invest. Ophthalmol. Vis. Sci. 45 (8), 2760–2766. 10.1167/iovs.03-1392 15277502

[B8] Ersahin T. Tuncbag N. Cetin-Atalay R. (2015). The PI3K/AKT/mTOR interactive pathway. Mol. Biosyst. 11 (7), 1946–1954. 10.1039/c5mb00101c 25924008

[B9] Fu D. Yu J. Y. Yang S. Wu M. Hammad S. M. Connell A. R. (2016). Survival or death: A dual role for autophagy in stress-induced pericyte loss in diabetic retinopathy. Diabetologia 59 (10), 2251–2261. 10.1007/s00125-016-4058-5 27475954 PMC5016562

[B10] Gerhardinger C. Dagher Z. Sebastiani P. Park Y. S. Lorenzi M. (2009). The transforming growth factor- pathway is a common target of drugs that prevent experimental diabetic retinopathy. Diabetes 58 (7), 1659–1667. 10.2337/db08-1008 19401417 PMC2699853

[B11] Grumati P. Coletto L. Sabatelli P. Cescon M. Angelin A. Bertaggia E. (2010). Autophagy is defective in collagen VI muscular dystrophies, and its reactivation rescues myofiber degeneration. Nat. Med. 16 (11), 1313–1320. 10.1038/nm.2247 21037586

[B12] Huang C. Zhang Y. Kelly D. J. Tan C. Y. R. Gill A. Cheng D. (2016). Thioredoxin interacting protein (TXNIP) regulates tubular autophagy and mitophagy in diabetic nephropathy through the mTOR signaling pathway. Sci. Rep. 6, 29196. 10.1038/srep29196 27381856 PMC4933928

[B13] Jacot J. L. Sherris D. (2011). Potential therapeutic roles for inhibition of the PI3K/Akt/mTOR pathway in the pathophysiology of diabetic retinopathy. J. Ophthalmol. 2011, 589813. 10.1155/2011/589813 22132311 PMC3205601

[B14] Joussen A. M. Poulaki V. Le M. L. Koizumi K. Esser C. Janicki H. (2004). A central role for inflammation in the pathogenesis of diabetic retinopathy. FASEB J. 18 (12), 1450–1452. 10.1096/fj.03-1476fje 15231732

[B15] Kameyama K. Motoyama K. Tanaka N. Yamashita Y. Higashi T. Arima H. (2017). Induction of mitophagy-mediated antitumor activity with folate-appended methyl-β-cyclodextrin. Int. J. Nanomedicine 12, 3433–3446. 10.2147/IJN.S133482 28496320 PMC5417668

[B16] Kern T. S. (2007). Contributions of inflammatory processes to the development of the early stages of diabetic retinopathy. Exp. Diabetes Res. 2007, 95103. 10.1155/2007/95103 18274606 PMC2216058

[B17] Kinuthia U. M. Wolf A. Langmann T. (2020). Microglia and inflammatory responses in diabetic retinopathy. Front. Immunol. 11, 564077. 10.3389/fimmu.2020.564077 33240260 PMC7681237

[B18] Liu M. M. Dong R. Hua Z. Lv N. N. Ma Y. Huang G. C. (2020). Therapeutic potential of Liuwei Dihuang pill against KDM7A and Wnt/β-catenin signaling pathway in diabetic nephropathy-related osteoporosis. Biosci. Rep. 40 (9), BSR20201778. 10.1042/BSR20201778 32914833 PMC7502694

[B19] Lorenzi M. Gerhardinger C. (2001). Early cellular and molecular changes induced by diabetes in the retina. Diabetologia 44 (7), 791–804. 10.1007/s001250100544 11508263

[B20] McCulloch D. L. Marmor M. F. Brigell M. G. Hamilton R. Holder G. E. Tzekov R. (2015). ISCEV Standard for full-field clinical electroretinography (2015 update). Doc Ophthalmol. 130 (1), 1–12. 10.1007/s10633-014-9473-7 25502644

[B21] Mizushima N. Komatsu M. (2011). Autophagy: renovation of cells and tissues. Cell 147 (4), 728–741. 10.1016/j.cell.2011.10.026 22078875

[B22] Mizutani M. Gerhardinger C. Lorenzi M. (1998). Muller cell changes in human diabetic retinopathy. Diabetes 47 (3), 445–449. 10.2337/diabetes.47.3.445 9519752

[B23] Nagai N. Deguchi S. Otake H. Hiramatsu N. Yamamoto N. (2017). Therapeutic effect of cilostazol ophthalmic nanodispersions on retinal dysfunction in streptozotocin-induced diabetic rats. Int. J. Mol. Sci. 18 (9), 1971. 10.3390/ijms18091971 PMC5618620 28906472

[B25] Noda T. (2017). Regulation of autophagy through TORC1 and mTORC1. Biomolecules 7 (3), 52. 10.3390/biom7030052 PMC5618233 28686223

[B26] Qin L. Pang L. Ou Y. Luo B. Luo Y. (2019). Research on clinical efficacy and mechanism of Mingmu Xiaomeng tablets combined with ranibizumab in treatment of wet age-related macular degeneration. Food Drug 21 (03), 222–226. 10.3969/j.issn.1672-979X.2019.03.014

[B27] Rabanal-Ruiz Y. Otten E. G. Korolchuk V. I. (2017). mTORC1 as the main gateway to autophagy. Essays Biochem. 61 (6), 565–584. 10.1042/EBC20170027 29233869 PMC5869864

[B28] Rabe E. Jaeger K. A. Bulitta M. Pannier F. (2011). Calcium dobesilate in patients suffering from chronic venous insufficiency: a double-blind, placebo-controlled, clinical trial. Phlebol. 26 (4), 162–168. 10.1258/phleb.2010.010051 21478142

[B29] Rosa M. D. Distefano G. Gagliano C. Rusciano D. Malaguarnera L. (2016). Autophagy in diabetic retinopathy. Curr. Neuropharmacol. 14 (8), 810–825. 10.2174/1570159x14666160321122900 26997506 PMC5333581

[B30] Roy S. Kern T. S. Song B. Stuebe C. (2017). Mechanistic insights into pathological changes in the diabetic retina. Am. J. Pathol. 187 (1), 9–19. 10.1016/j.ajpath.2016.08.022 27846381 PMC5225303

[B31] Saeedi P. Petersohn I. Salpea P. Malanda B. Karuranga S. Unwin N. (2019). Global and regional diabetes prevalence estimates for 2019 and projections for 2030 and 2045: Results from the international diabetes federation diabetes atlas, 9th edition. Diabetes Res. Clin. Pract. 157, 107843. 10.1016/j.diabres.2019.107843 31518657

[B32] Shafabakhsh R. Aghadavod E. Mobini M. Heidari‐Soureshjani R. Asemi Z. (2019). Association between microRNAs expression and signaling pathways of inflammatory markers in diabetic retinopathy. J. Cel. Physiol 234 (6), 7781–7787. 10.1002/jcp.27685 30478931

[B33] Simó R. Ballarini S. Cunha-Vaz J. Ji L. Haller H. Zimmet P. (2015). Non-traditional systemic treatments for diabetic retinopathy: an evidence-based review. Curr. Med. Chem. 22 (21), 2580–2589. 10.2174/0929867322666150520095923 25989912 PMC4997935

[B34] Simó R. Hernández C. (2014). Neurodegeneration in the diabetic eye: new insights and therapeutic perspectives. Trends Endocrinol. Metab. 25 (1), 23–33. 10.1016/j.tem.2013.09.005 24183659

[B35] Simó R. Hernández C. (2012). Neurodegeneration is an early event in diabetic retinopathy: therapeutic implications. Br. J. Ophthalmol. 96 (10), 1285–1290. 10.1136/bjophthalmol-2012-302005 22887976

[B36] Sinclair S. H. Schwartz S. S. (2019). Diabetic retinopathy-an underdiagnosed and undertreated inflammatory, neuro-vascular complication of diabetes. Front. Endocrinol. 10, 843. 10.3389/fendo.2019.00843 PMC6923675 31920963

[B37] Solomon S. D. Chew E. Duh E. J. Sobrin L. Sun J. K. VanderBeek B. L. (2017). Diabetic retinopathy: a position statement by the American diabetes association. Dia Care 40 (3), 412–418. 10.2337/dc16-2641 PMC5402875 28223445

[B38] Song P. Yu J. Chan K. Y. Theodoratou E. Rudan I. (2018). Prevalence, risk factors and burden of diabetic retinopathy in China: a systematic review and meta-analysis. J. Glob. Health 8 (1), 010803. 10.7189/jogh.08.010803 29899983 PMC5997368

[B39] Tewari D. Patni P. Bishayee A. Sah A. N. Bishayee A. (2019). Natural products targeting the PI3K-Akt-mTOR signaling pathway in cancer: A novel therapeutic strategy. Semin. Cancer Biol. S1044-579X (19), 30405. 10.1016/j.semcancer.2019.12.008 31866476

[B40] Toft-Kehler A. K. Skytt D. M. Svare A. Lefevere E. Van Hove I. Moons L. (2017). Mitochondrial function in Müller cells - does it matter?. Mitochondrion 36, 43–51. 10.1016/j.mito.2017.02.002 28179130

[B41] Wang Y. Lu Y.-h. Tang C. Xue M. Li X.-y. Chang Y.-p. (2019). Calcium dobesilate restores autophagy by inhibiting the VEGF/PI3K/AKT/mTOR signaling pathway. Front. Pharmacol. 10, 886. 10.3389/fphar.2019.00886 31447680 PMC6696883

[B42] Wang Y. Pang L. Ou Y. Yuan Y. Li H. Qiu B. (2014). Therapeutic effect of Xiaomeng tablets for dry age-related macular degeneration patients with syndrome of qi-yin deficiency and phlegm blended with blood stasis. J. New Chin. Med. 46 (07), 133–135. 10.13457/j.cnki.jncm.2014.07.063

[B43] Wang Y. Wang J. Qiu B. Zhang M. (2018). Effect of xiaomengling on the TNF-a and JAK2/STAT5 pathway in the retina of diabetes mellitus rats. Chin. J. Optom. Ophthalmol. Vis. Sci. 20 (08), 492–497+512. 10.3760/cma.j.issn.1674-845X.2018.08.007

[B44] Wilkinson C. P. Ferris F. L. Klein R. E. Lee P. P. Agardh C. D. Davis M. (2003). Proposed international clinical diabetic retinopathy and diabetic macular edema disease severity scales. Ophthalmol. 110 (9), 1677–1682. 10.1016/S0161-6420(03)00475-5 13129861

[B45] Wu M.-Y. Yiang G.-T. Lai T.-T. Li C.-J. (2018). The oxidative stress and mitochondrial dysfunction during the pathogenesis of diabetic retinopathy. Oxid. Med. Cell Longev. 2018, 3420187. 10.1155/2018/3420187 30254714 PMC6145164

[B46] Yan X. Qin L. (2019). Therapeutic effect of Mingmu Xiaomeng tablets combined with ranibizumab on wet age-related macular degeneration. Clin. Res. Pract. 4 (34), 137–138. 10.19347/j.cnki.2096-1413.201934059

[B47] Yao L. Wang J. Tian B.-Y. Xu T.-H. Sheng Z.-T. (2017). Activation of the nrf2-ARE signaling pathway prevents hyperphosphatemia-induced vascular calcification by inducing autophagy in renal vascular smooth muscle cells. J. Cel. Biochem. 118 (12), 4708–4715. 10.1002/jcb.26137 28513870

[B48] Yu J. S. L. Cui W. (2016). Proliferation, survival and metabolism: the role of PI3K/AKT/mTOR signalling in pluripotency and cell fate determination. Development 143 (17), 3050–3060. 10.1242/dev.137075 27578176

[B49] Zhan W. Pang L. Deng F. Qiu B. (2012). Clinical research of Xiaomengling Tablet on rhegmatogenous retinal detachment patients after vitreoretinal surgery. China J. Chin. Ophthalmol. 22 (01), 20–22. 10.13444/j.cnki.zgzyykzz.002925

[B50] Zhang C. Qiu B. Zhang M. (2011). Inhibitory effec of xiao Meng ling decoction on retinal angiogenesis in diabetic rats. Traditional Chin. Drug Res. Clin. Pharmacol. 22 (06), 620–623. 10.19378/j.issn.1003-9783.2011.06.011

[B51] Zhang Q. Xiao X. Zheng J. Li M. Yu M. Ping F. (2018). Compound danshen dripping pill inhibits retina cell apoptosis in diabetic rats. Front. Physiol. 9, 1501. 10.3389/fphys.2018.01501 30405447 PMC6207599

[B52] Zhang X. Liu W. Wu S. Jin J. Li W. Wang N. (2015). Calcium dobesilate for diabetic retinopathy: a systematic review and meta-analysis. Sci. China Life Sci. 58 (1), 101–107. 10.1007/s11427-014-4792-1 25528255

[B53] Zong H. Ward M. Madden A. Yong P. H. Limb G. A. Curtis T. M. (2010). Hyperglycaemia-induced pro-inflammatory responses by retinal Müller glia are regulated by the receptor for advanced glycation end-products (RAGE). Diabetologia 53 (12), 2656–2666. 10.1007/s00125-010-1900-z 20835858

[B54] He Y. Dan Y. Gao X. Huang L. Lv H. Chen J. (2020). DNMT1-mediated lncRNA MEG3 methylation accelerates endothelial-mesenchymal transition in diabetic retinopathy through the PI3K/AKT/mTOR signaling pathway. Am. J. Physiol. Endocrinol. Metab. 320 (3), E598–E608. 10.1152/ajpendo.00089.2020 33284093

