# Supplementary figures and images for: Mingmu Xiaomeng Tablets Restore Autophagy and Alleviate Diabetic Retinopathy by Inhibiting PI3K/Akt/mTOR Signaling
Source: Front Pharmacol. 2021 Apr 13;12:632040. doi: 10.3389/fphar.2021.632040 (PMC8077025; doi:10.3389/fphar.2021.632040)

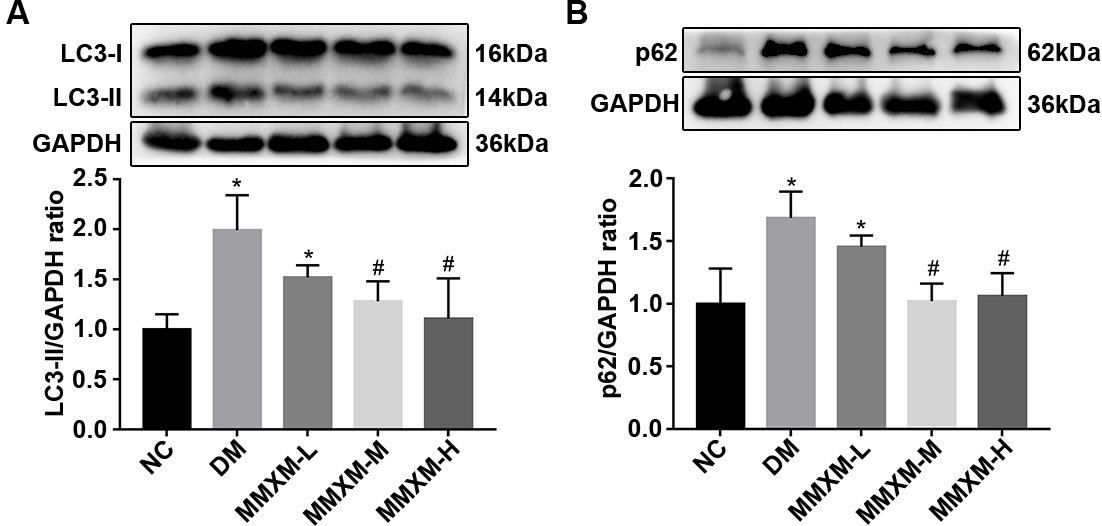

Supplement: Supplementary file 1 [file image1.tif]
